# Core body temperature speeds up temporal processing and choice behavior under deadlines

**DOI:** 10.1038/s41598-019-46073-3

**Published:** 2019-07-11

**Authors:** Leendert van Maanen, Robbert van der Mijn, Maurice H. P. H. van Beurden, Linsey M. M. Roijendijk, Boris R. M. Kingma, Steven Miletić, Hedderik van Rijn

**Affiliations:** 1 Department of Psychology, University of Amsterdam, Amsterdam, The Netherlands; 2 Department of Psychology, University of Groningen, Groningen, The Netherlands; 3 Netherlands Organization for Applied Scientific Research, Unit Defense Safety and Security, Department of Training and Performance Innovations, Soesterberg, The Netherlands

**Keywords:** Human behaviour, Decision

## Abstract

Evidence suggests that human timing ability is compromised by heat. In particular, some studies suggest that increasing body temperature speeds up an internal clock, resulting in faster time perception. However, the consequences of this speed-up for other cognitive processes remain unknown. In the current study, we rigorously tested the speed-up hypothesis by inducing passive hyperthermia through immersion of participants in warm water. In addition, we tested how a change in time perception affects performance in decision making under deadline stress. We found that participants underestimate a prelearned temporal interval when body temperature increases, and that their performance in a two-alternative forced-choice task displays signatures of increased time pressure. These results show not only that timing plays an important role in decision-making, but also that this relationship is mediated by temperature. The consequences for decision-making in job environments that are demanding due to changes in body temperature may be considerable.

## Introduction

Accurate decision making is crucial for survival. A decision to brake in response to the brake lights of the preceding car may constitute the difference between an accident and a safe arrival at your destination. Similarly, in demanding job environments – for example, active duty of military personnel – a timely and correct decision can make the difference between life and death. In both these examples, it is clear that the passing of time plays a role: The task at hand for the motorist or the soldier is not only to choose between two (or more) courses of action, but to do so within a certain time window.

**Figure 1:**
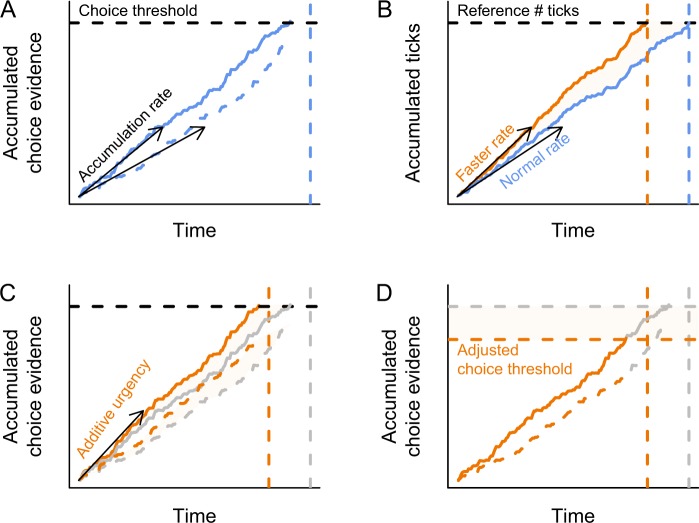
Hypothesized effects of core temperature on behavior. (**A**) In a choice task, evidence is accumulated for two response alternatives (solid and dashed lines) until a choice threshold is reached (horizontal dashed line). Participants are instructed to respond before a deadline (vertical dashed line). (**B**) We hypothesize that an internal pacemaker emitting pulses at regular intervals speeds up when core temperature is higher (The speed up is indicated by the orange lines, relative to the blue line). This will result in an underestimation of previously learned temporal intervals (vertical dashed orange line), and consequently in a perceived change of a choice deadline. The altered perception of the response deadline results in time pressure, which may be implemented by an additional temporal signal that is added to the perceptual evidence (**C**) or (**D**) by decreasing a choice threshold, resulting in commitment to a response based on less information.

Possibly for this reason, decision making under time pressure has been studied extensively. The dominant theoretical framework assumes that decision makers accumulate evidence for the various choice alternatives at hand, until they accumulate enough evidence to commit to one option (Fig. 1A)^1–3^. One major finding in the decision-making field is that decision makers sacrifice accuracy of responding for response speed when pressed for time^4–7^. Several questions about how such a speed-accuracy trade-off is achieved remain unanswered. Firstly, different cognitive mechanisms are hypothesized that all result in a speed-accuracy trade-off, but achieve it in different ways^8–15^. Secondly, increasing response speed at the expense of accuracy at some point during a decision process requires a sense of the passing of time. Whether temporal processing is therefore required for speed-accuracy trade-offs in decision making remains unknown^16^.

The current study focusses on this second question. Humans are able to keep track of time with remarkable accuracy, albeit with varying precision (e.g.^17–19^). Although theories of time perception vary in the specific cognitive mechanisms or the neural implementations, the standard theory assumes a pacemaker that emits ticks at a regular rate, which are read out by an accumulator (Fig. 1B)^17,20–23^. The accumulator can be reset if an event happens that marks the beginning of an interval. To estimate a particular duration, the number of ticks is compared to a number stored in memory as a reference^24^. If the number of ticks matches the reference, then the interval has passed.

Because a tradeoff between response accuracy and speed seems to require a sense of time, we hypothesized that the same mechanisms that allow people to accurately time events also determine how people adjust their decision-making behavior in the face of time pressure. To study this, we experimentally induced a bias in the estimates that participants reproduced from a previously learned temporal interval. This bias was induced by elevating the core temperature of participants, which is believed to speed up the internal clock, resulting in overestimation of how much time has passed and thus shorter temporal reproductions (which we will refer to as *underproduction)*^25,26^. The core temperature was elevated by immersion (from the neck down) in a hot tub with a water temperature of 38 °C, meant to induce passive hyperthermia (henceforward the Warm condition). Behavior in the hot tub was compared to a control condition in which the water temperature did not induce hyperthermia (the Neutral condition). Moreover, we measured behavior at two measurement moments (Begin and End of the tub sessions), to understand whether immersion in hot water or the change in body temperature would best explain changes in time estimation.

We reasoned that if a speed-up of the internal clock also plays a role in decision making under time pressure, then we should observe changes in behavior in a choice task that reflect the underproduction. This reasoning leads to the hypothesis that higher core temperature results in underestimation of deadlines in choice behavior. Consequently, participants would have to adjust their decision-making behavior to be able to respond before the perceived – underestimated – deadline. Following the dominant theories about speed-accuracy trade-off behavior, such an underestimation of the deadline would result in either an urgency signal involved in the accumulation of evidence for each choice alternative (“additive urgency component” in Fig. 1C)^8,27,28^, or a lower choice threshold (Fig. 1D)^5,29,30^.

**Figure 2:**
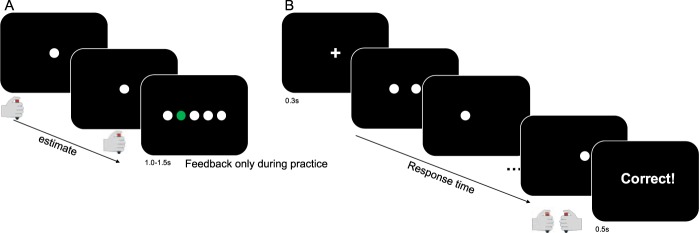
Experimental design. The participants first performed a time estimation task (**A**). The second task involved a two-alternative forced choice task (**B**).

In the time estimation task, participants were asked to reproduce a prelearned interval by two consecutive button presses (Fig. 2A)^31,32^. The choice task used a fast-paced expanded judgment paradigm^33–35^ involving two flickering squares. The participant was instructed to choose the circle with the higher flicker rate (Fig. 2B). Participants were instructed to try to make no mistakes, however they should also respond within a deadline that was equal to the prelearned interval from the time estimation task.

## Results

### Descriptives

**Figure 3:**
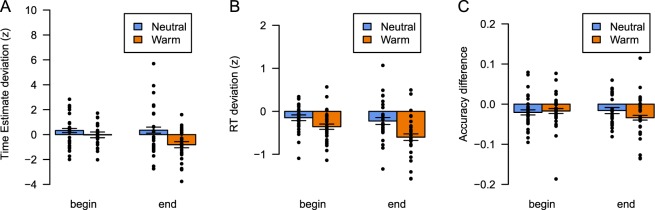
Results show that increased core temperature affects timing and choice behavior. (**A**) Time estimation relative to the baseline measurement decrease at the end of the Warm condition (**B**) Choice response time (RT) relative to the baseline measurement is decreased, and particularly in the Warm condition. (**C**) Proportion correct responses (Accuracy) as a difference relative to the baseline measurement is decreased. Error bars indicate within-subject standard errors of the mean, dots indicate individual estimates.

Consistent with our hypotheses, we found that participants in the Warm condition provided shorter estimates of the temporal interval at the end of the hot tub session, relative to a baseline out-of-tub measurement (Fig. 3A and Table 1). A comparison between a regression model that included the interaction between water temperature (Neutral vs. Warm) and measurement moment (Begin vs. End of the tub session) and a model without this interaction showed that it was indeed preferred (the difference in Bayesian Information Criterion (BIC^36^, see Materials and Methods) is in favor of the more complex model, ΔBIC = 338). This suggests that not the water temperature, but the increase in body temperature throughout the session induces the overestimation of how much time has passed, resulting in shorter reproductions of the prelearned temporal interval.

**Table 1:** Coefficients of the preferred regression model for the interval timing task.

	*β*(se)	*t*(26)	*p*
Neutral	0.34 (0.32)	1.06	0.30
Warm	−0.41 (0.17)	−2.42	0.023
End	0.19 (0.10)	1.96	0.061
Warm × End	−0.21 (0.07)	−3.15	0.004

The change in body temperature also influenced choice behavior. Response times were faster in the immersion conditions relative to the baseline measurement, but increasingly so for the Warm condition, and even more at the end of the session (Fig. 3B and Table 2). A comparison between a regression model that included the interaction between water temperature (Neutral vs. Warm) and measurement moment (Begin vs. End) and a model without the interaction showed again a preference for the model that included the interaction (ΔBIC = 313), although the regression weights for the interaction (Warm x End) did not deviate from zero. In terms of choice accuracy, the results were less clear. A logistic regression model that includes the interaction between water temperature and measurement moment is not preferred over a model without the interaction (Fig. 3C and Table 3, ΔBIC = −6.9). However, an additional model in which we tested whether the Warm x End design cell deviated from the others was preferred (ΔBIC = 98 over the best model) and showed a significant drop in accuracy for this design cell relative to baseline (*β* = −0.10 (0.05); z(26) = −2.2; p = 0.029).This ambiguous result may be due to the discrete nature of the accuracy measure. In our view, this calls for an analysis in which response times and accuracy are jointly investigated, which we will report on in the next section.

**Table 2:** Coefficients of the preferred regression model for the choice task (response times).

	*β*(se)	*t*(26)	*p*
Neutral	−0.19 (0.07)	−2.74	0.01
Warm	−0.48 (0.08)	−6.11	<0.001
End	−0.08 (0.02)	−3.54	0.002
Warm × End	−0.04 (0.03)	−1.41	0.17

**Table 3:** Coefficients of the preferred logistic regression model for the choice task (accuracy).

	*β*(se)	z(26)	*p*
Baseline	2.64 (0.43)	6.09	<0.001
Neutral	−0.81 (0.35)	−2.30	0.02
Warm	−0.86 (0.35)	−2.48	0.01
End	0.02 (0.02)	0.78	0.44

An important consequence of our hypothesis is that individual differences in people’s ability to accurately estimate the deadline should be reflected in choice behavior. Specifically, we predicted that the observed speed up in choice response times with body temperature was larger for participants that more strongly underproduced the temporal interval. Such a relationship would reflect the idea that underproduction is a proxy for the underestimation of the deadline in the choice task, which should affect choice response time. A similar reasoning would hold for the change in response accuracy with body temperature. Individuals who underproduce the temporal interval more should also make more errors.

**Figure 4:**
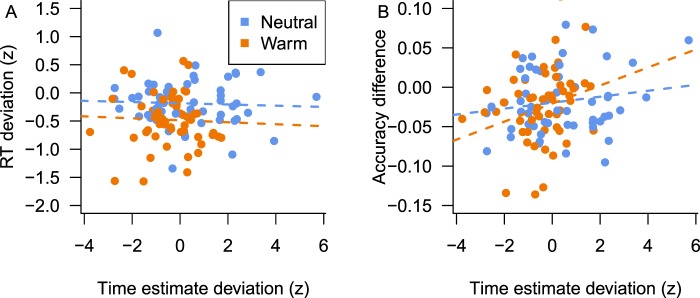
Changes in behavior across tasks. (**A**) Time estimation relative to baseline does not predict choice response time (RT) relative to baseline. (**B**) Time estimation relative to baseline predicts accuracy in the choice task (difference with baseline).

Contrary to this hypothesis, we did not find a correlation between time estimation (relative to baseline) and mean response times (relative to baseline), in either temperature condition (Fig. 4A, repeated-measures ANCOVA, F_estimate_ < 1; F_condition_(1, 24) = 6.9, p = 0.016; F_estimate × condition_ < 1). However, the hypothesis was supported by the positive correlation between time estimation (relative to baseline) and the accuracy (change from baseline) (Fig. 4B, repeated-measures ANCOVA, F_estimate_(1, 52) = 4.5, p = 0.044, r = 0.20; F_condition_ < 1; F_estimate × condition_ < 1). That is, the more participants underestimate the temporal interval (in either condition), the more errors they make in the choice task. It is important to note that this effect was not driven by individual differences in the baseline measurements of both tasks, since these did not show any relationship (Fig. S1, all correlation *t*-values < 1).

### Computational modeling

We hypothesized that the observed behavioral changes in the choice task would be best explained by assuming that participants felt an increased urgency to respond quickly when their core temperature was elevated. This would reflect a shifted internal representation of the deadline before responses ought to be given. Such an increased urgency could be implemented by a temporal signal that adds to the stimulus-driven accumulation of evidence^27,28,37^ (Fig. 1C). In the computational model described below, this is identical to a so-called collapsing bound^8,9^ (for a proof see ref. 38). Alternatively, we hypothesized that a near-optimal strategy to respond before the perceived deadline would be to lower the choice threshold (Fig. 1D). This would be consistent with standard models of instructed speed-accuracy trade-offs (e.g. ref. 39), but would not guarantee a response before the deadline.

We fit three instances of an evidence accumulation model to the choice RT and accuracy data (the Linear Ballistic Accumulator model (LBA^40^), see Materials and Methods for details). The first model assumed that the differences in response time and accuracy between the design cells were completely driven by different additive urgency signals between design cells. In the LBA model, differences in urgency signals are implemented by different evidence accumulation rates. Conversely, the second model assumed that the differences in behavior between design cells were completely driven by different threshold values. The third model assumed that both these properties could vary across design cells.

**Figure 5:**
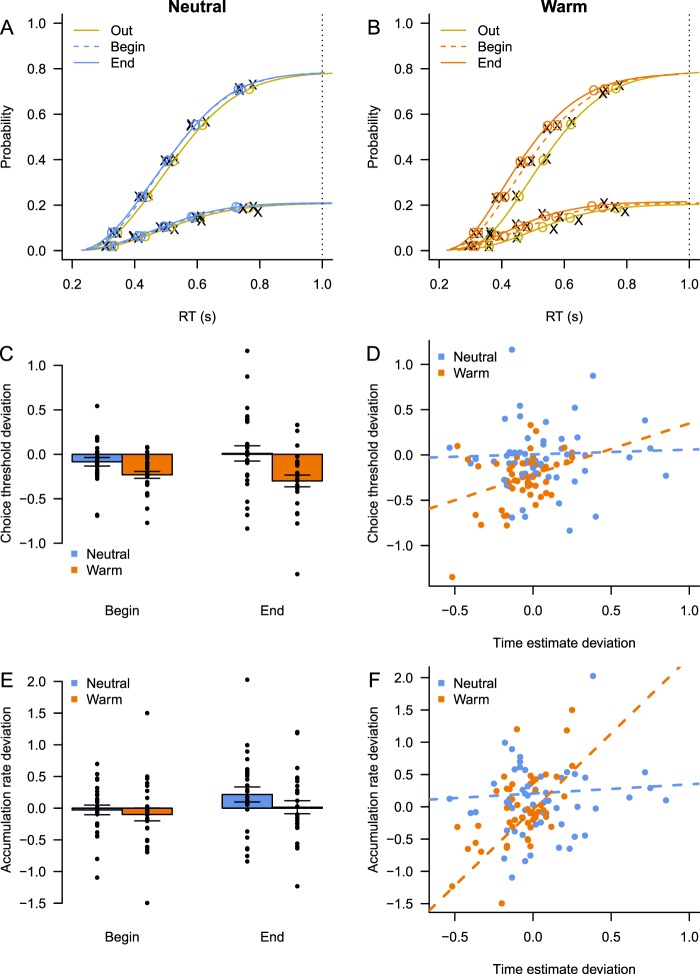
Computational modeling results. (**A**) Aggregate fit of the LBA model on the data from the choice task, Neutral condition. The figure shows defective cumulative density functions^40^ of the correct and incorrect responses in the data (the X symbols), overlaid with the predictions of the LBA model (the O symbols and lines), separately for each measurement moment. The dotted vertical line represents the response deadline. (**B**) Same as A, but for the Warm condition. (**C**) Choice thresholds (compared to baseline) change in the Warm condition. (**D**) The mean estimates in the timing task (compared to baseline) predict changes in threshold setting in the choice task. (**E**) Accumulation rate (compared to baseline) differ between measurement moments. (**F**) Changes in the mean estimate in the timing task predict changes in the evidence accumulation rate. Error bars indicate within-subject standard errors of the mean, dots indicate individual estimates.

We found that this last model best explained the observed speed up in responses and decrease in accuracy. This model fits the distribution of the choice data well. Figure 5A,B show the cumulative response time distributions for each design cell, separately for correct and incorrect responses, and scaled for the proportion of correct and incorrect responses (so-called defective cumulative density functions^40^). The model’s prediction using the best fitting parameters (Table S1) closely overlaps with the data, particularly for the response times of correct responses. Moreover, this model had a better balance between goodness of fit and model flexibility according to a Bayesian Predictive Information Criterion (BPIC^41^), than a model that only allowed thresholds to vary between conditions (ΔBPIC = 166). It was also preferred over a model that only assumed different urgency signals (ΔBPIC = 703). Finally, this model’s predictions with respect to the relationship between accuracy and response time were consistent with the data (Supplementary Analysis 1).

Analyzing the parameter estimates of the winning LBA model showed that the choice threshold systematically decreased in the Warm condition (Fig. 5C, F_cond_(1, 26) = 10.4, p = 0.003; F_moment_(1, 26) = 0.05, p = 0.82; F_moment × condition_(1, 26) = 1.32, p = 0.262), consistent with our alternative hypothesis that increases in core temperature decrease choice thresholds. Moreover, the amount of decrease of the choice threshold in the Warm condition correlated positively with the underestimation of the temporal interval (Fig. 5D, repeated-measures ANCOVA, F_time_(1, 52) = 5.4, p = 0.029, r = 0.16; F_condition_(1, 52) = 5.4, p = 0.029; F_time × condition_(1, 52) = 2.5, p = 0.13), in both temperature conditions. The main effect of time estimation on choice threshold was strongest in the Warm condition (r = 0.41, t(52) = 3.3, p = 0.002).

In contrast, although the formal comparison between LBA models showed that drift rates ought to vary between all conditions, this did not result in a systematic change of rate of evidence accumulation across temperature conditions, relative to baseline (Fig. 5E, repeated-measures ANOVA, F_condition_(1, 26) = 1.64, p = 0.21; F_moment_(1, 26) = 3.16, p = 0.087; F_moment × condition_(1, 26) = 0.3, p = 0.59). We did however observe a positive correlation between accumulation rates relative to baseline and the estimation of the temporal interval (Fig. 5F, repeated-measures ANCOVA, F_time_(1, 52) = 2.3, p = 0.14; F_condition_(1, 52) < 1; F_time × condition_(1, 52) = 10.0, p = 0.004). The interaction effect was predominantly driven by an effect of time estimation on accumulation rate in the Warm condition (r = 0.56, t(52) = 4.8, p < 0.001). It is important to realize that the direction of this correlation is opposite to what we hypothesized. That is, participants do not *increase* their evidence accumulation rate when underproducing a temporal interval – i.e., when their internal clock speeds up – but show a decrease in the accumulation rate.

## Discussion

In the current paper we tested and confirmed a number of hypotheses. Firstly, we confirmed that changes in core temperature induce faster temporal processing. A small number of studies have investigated the existence of a temperature-sensitive internal clock^25,26,42^. These studies suggested that core temperature speeds up an internal clock, resulting in shorter reproductions of temporal durations^25^. Our results support this interpretation.

In the absence of feedback on their performance, our participants tended to underproduce a target temporal duration, a finding consistent with previous studies^43^. However, during immersion in a hot tub, leading to an increased core body temperature, these underproductions became smaller, indicating a speed up of the internal clock. Here, the effect of arousal caused by heat distress cannot be ignored. As core temperature rose during immersion, subjects also had an increased heart rate (Supplemental Fig. S2). The effects of arousal on temporal reproduction have been found in the context of emotional arousal^44^ and physiological arousal^26^ as well. Our findings are in line with these previous studies. However, most of these studies focussed on temporal intervals that were longer (>10 s), studied temporal processing indirectly^45,46^, or did not control for possible confounding factors such as time of day^45,47,48^ or fatigue^26,42^.

The second hypothesis that we confirmed was that immersion in a hot tub reduced response times as well as accuracy in a typical choice task, and that variations in this behavior related to variations in participant’s changes in timing ability. Specifically, we found that larger underproductions of a temporal interval were associated with more errors. This suggests that the same timing mechanisms required for explicitly reproducing a temporal interval, are also required for estimating the passing of time during a choice task that involves a deadline. If the timing mechanisms of the two tasks would have been unrelated, we would not have observed that the behavioral changes correlated.

The third hypothesis that we confirmed was that the best way to describe the choice behavior was through an evidence accumulation model that assumed that participants decreased their choice threshold when their body temperature increased. This suggests a specific mechanism to deal with the perceived change in response deadline. Decision makers seem to adjust a static choice threshold to ensure that the majority of the responses (but not all) are before the deadline. The alternative strategy for which we did not find evidence is that participants augment the accumulation of evidence with an urgency signal to speed up responses.

The normative optimal way of responding accurately before a deadline, is by adopting a declining or collapsing choice threshold^9,49^. This means that over time, the critical amount of information required to commit to a decision decreases. Such a strategy maximizes rewards over a series of choices, if correct choices are rewarded by a certain gain, but time is associated with a certain cost. Whether decision makers actually adopt this optimal strategy is debated however, with some researchers showing evidence against such optimal behavior in experimental data^10,11,15^, but others arguing that in specific cases the data has signatures of optimal decision making^28,33,37,38^.

Although a decrease in choice threshold to accommodate a perceived earlier deadline - as we found – is a suboptimal strategy from a normative point of view, recent computational analyses suggest that such a strategy is close to optimal in many scenarios. Boehm *et al*.^50^ argued that the expected reward in a sequence of choices was nearly identical for an (optimal) time-dependent threshold and the best static threshold. Only in cases where the stimulus was extremely noisy, a larger difference in rewards between the strategies was found. Given that these extreme stimulus conditions did not hold in our case, a change in static choice threshold seems consistent with previous literature.

Another component of decision making before a temporal deadline that we did not discuss, is the uncertainty in the temporal reproduction required for estimating the deadline. If participants are certain about the temporal location of the deadline, and want to respond before the deadline, then they will adjust their choice behavior accordingly. This component was explicitly addressed in a separate study^38^. In that study, we found that indeed timing uncertainty (as indexed by for example variability in the topDDM model of time production^51,52^) predicts individual response thresholds. Following Frazier and Yu and Karsilar *et al*.^9,15^ we reasoned that this adjustment reflected participants’ reward optimization. The specific reward scheme in that study meant that missing the deadline was costlier than committing to an incorrect choice, and hence uncertainty about the temporal location of the deadline would be reflected in faster decision commitment combined with an increased error rate. The current study follows up on this result by showing that an experimentally induced deadline shift complements these observed individual variations in choice threshold.

We also found that participants whose performance in the timing task decreased the most (in terms of an underestimation of the temporal interval), also showed the strongest decrease in evidence accumulation (Fig. 5F). This positive correlation cannot be explained by our initial hypothesis about the relationship between additive urgency and timing ability. That hypothesis implies that the urgency component of evidence accumulation would *increase* when participants produce shorter durations in the timing task, suggesting a *negative* correlation. In the LBA model, the evidence accumulation parameter that was estimated can be interpreted as a timing-related urgency signal, or as a reflection of the speed with which the stimulus is processed (i.e., it is the sum of these components). A possible explanation for the observed positive correlation is therefore that the speed of stimulus processing is affected by the physical demands of the experiment. Particularly in the Warm condition, participants found the experiment quite hard, possibly resulting in loss of attention or focus. Indeed, previous work has also shown that the speed of stimulus processing decreases when attention decreases^53–55^, consistent with a positive correlation between the rate of evidence accumulation and timing accuracy.

So far, we have entertained the theory that decision-makers have adjusted their choice behavior in response to a misperception of the temporal deadline. This theory assumes that only timing is influenced by a change in core body temperature, and not decision making per se. An alternative explanation is that both timing as well as “threshold setting” are affected by changes in body temperature in a similar way. This alternative theory is supported by meta-analyses that suggest that extreme temperatures have detrimental effects on a range of cognitive tasks^56,57^. When the temperature stressors are intense and exposure is long, as in our experiment, cognitive functioning as a whole deteriorates. In contrast, small increases in body temperature were found to have a positive effect on cognitive performance and alertness^58^.

This explanation is also supported by the observation that the timing and choice tasks share a neural architecture. Specifically, the striatum has been shown to reflect threshold settings in choice tasks in which participants are instructed to switch between fast responding and accurate responding^39,59–62^ or to respond before a deadline^33,63^. At the same time, the striatum is involved in the speed of the internal clock^20,64,65^. Particularly, pharmacological interventions in rodents have shown that influencing the dopaminergic uptake of the striatum speeds up the internal clock^66,67^. One intriguing explanation of our results is then that the change in body temperature that causes the internal clock to speed up, does so by increasing synaptic dopamine in the striatum. This would result in a faster internal clock, but at the same time would cause changes in a choice threshold. A number of studies indeed hypothesize a role of dopamine uptake in the adjustment of response thresholds (but see^30,68–70^). Whether the relationship between timing and decision making appears on a neurobiological level, or whether this relationship is a consequence of cognitive control in one task in response to another, is an exciting new avenue for future work.

## Materials and Methods

### Participants

The study was approved by the local ethics committee of the Netherlands Organization for Applied Scientific Research, Unit Defense Safety and Security (TC-nWMO, registration number 2017-023), and performed in accordance with relevant guidelines and regulations. 29 male (mean age 23, mean body mass index BMI 22.9) participants enrolled in the experiment for a monetary reward. We included only male participants because the baseline temperature of females is more likely to differ between testing days, and their thermophysiological response differs substantially from males^71^. The participants were recruited through various channels, including the participant pool of University of Groningen, the participant pool of the institute where the experiments took place, and a Facebook group for paid participation in research experiments. The size of this sample was limited by the availability of the hot tubs and the complexity of the counterbalancing scheme. Prior to the experiment, all participants were screened for contra-indications to participate in a passive hyperthermia experiment by a medical doctor, including medicine usage, a history of syncope, and extreme BMI (<18 or >28). All participants signed an informed consent form. Two participants were excluded because of not following instructions in the timing task (duration estimations <250 ms).

### Experimental design

Participants performed an interval timing task and a two-alternative forced choice (2AFC) task that were designed to assess cognitive performance when core body temperature increased. The manipulation of core body temperature was operationalized by immersion of the participants in a hot tub. The water temperature was 36 °C in the Neutral condition, and the water temperature was 38 °C in the Warm condition to induce passive hyperthermia. Based on a pilot procedure, 38 °C was found as the temperature that best balanced the comfort of the participant (not too hot) with the rise of the core body temperature^72^.

In the interval timing task^31,32^, the participants experienced a 1 s interval by showing a circle that changed color every 1 s. Subsequently, they were asked to press a button when a fixation cross appeared on the middle of the screen, followed by another button press exactly after the experienced interval (Fig. 2A). The next trial started after an inter-trial interval that was sampled from a uniform distribution between 1 s and 1.5 s. The 2AFC task used a fast-paced expanded judgment paradigm^33–35^ in which two flickering white circles on a black background appeared next to each other on the screen. The flickering was governed by samples from two binomial distributions with different rates (0.7 for the target, 0.3 for the foil, randomly assigned to left or right response on each trial). The participant was instructed to choose the square with the higher flicker rate (Fig. 2B). Participants were instructed to try to make no mistakes, however they should also respond within a deadline of 1 s. After 1 s, feedback was given on the screen about their performance (‘correct’, ‘incorrect’, ‘too slow’).

### Procedure

All participants visited the lab on two occasions, separated by at least one day. The time of day was kept constant per participant, and the order of the experimental conditions (Neutral or Warm) was counterbalanced across conditions, such that half of the participants was in the Neutral condition on the first occasion, and the other half was in the Warm condition on the first occasion. After a short briefing on the nature of the experimental manipulation and signing the consent form, participants ingested a capsule for measurement of core temperature (e-Celsius Performance, BodyCap, Caen, France), were equipped with a heart rate monitor (Polar), and changed into their bathing suit. Next, participants practiced the tasks. They performed 50 trials of the interval timing task and 50 trials of the expended judgment task to get familiarized with the task. During the interval timing task, the participants received feedback on every trial about the accuracy of their estimation (Fig. 2A), which enabled them to learn an accurate representation of the temporal interval (Supplementary Fig. S3).

The participants performed both tasks three times per occasion: once as a baseline test and twice in a hot tub. At baseline, the mean core body temperature was respectively 37.1 °C (se = 0.04 °C) and 37.0 °C (se = 0.08 °C) before the Neutral and Warm immersion, indicating no difference in body temperature prior to the experimental manipulation. After 20 min of immersion, the participants performed the tasks (referred to as *Begin* measurement moment). After 60 min of immersion, or as soon as their core body temperature reached 38.5 °C, participants performed the tasks again (the *End* measurement moment). After the *End* measurement the participants exited the tub. Heart rate and core body temperature were monitored for at least 20 minutes after the experiment ended. Participants were dismissed when their core body temperature had decreased below 38 °C and they reported no complaints.

#### Manipulation check

Core temperature as well as heart rate increased in participants in the Warm condition (Fig. S2). Specifically, we observed main effects of Condition (ANOVA on temperature: F(1, 18) = 247, p < 0.001; ANOVA on Heart rate: F(1, 25) = 143, p < 0.001) and measurement moment (temperature: F(2,52) = 121, p < 0.001; Heart rate: F(2, 54) = 118, p < 0.001), as well as an interaction (temperature: F(2, 35) = 145, p < 0.001; Heart rate: F(2, 49) = 84.2, p < 0.001, note that the degrees of freedom differ because of equipment failure in some blocks). There were no differences in core temperature and heart rate between conditions at the baseline out-of-tub measurement (paired t-tests, all *t*-values < 1.2). Moreover, participants were able to accurately reproduce the 1 s interval in the practice block in both sessions (Fig. S3).

#### Data analysis

We compared – for each dependent measure – a generalized mixed effects regression model with an interaction between the two factors of interest (Water temperature – Neutral vs. Warm – and measurement moment – Begin vs. End) and one model without the interaction, using a Bayesian Information Criterion^36^ to control for the additional degree-of-freedom in the interaction model. The rationale is that if the core temperature that is induced through the immersion in the hot tub affects behavior, it should do so most at the end of the Warm session. To control for differences in the baseline measurement, the continuous variables (time estimations, response times), were expressed as a z-score relative to baseline. Because z-scoring the nominal accuracy variable is meaningless, we included the proportion of correct responses during the baseline measurement as a covariate to control for individual differences in baseline behavior. Response times were log-transformed to reduce the impact of extreme data points and thus improve precision with which the standard error of the mean is estimated by ensuring more normally distributed residuals e.g.^73,74^, but see^75^. We fit generalized linear mixed effects models with random participant intercepts and slopes per condition^76,77^. If the fixed effects in a particular model included an interaction, then the random effects included an interaction as well.

To study the correlations across tasks, and between the model parameters (see below) and the behavioral parameters, we performed analysis of covariance. This method takes the dependence between repeated-measure into account, and is preferred over linear mixed effects in case of limited repeated measures^77^. All measures were computed as the difference between the baseline measurement and factor level of interest. For the same reason of limited repeated measures, we also used analysis of (co)variance to study the systematic fluctuations of computational model parameters across conditions (see next section). Because the computational models estimates individual parameters in a hierarchical model, it underestimates the between-participant variability in the parameters (i.e., shrinkage). This entails that the correlations we report are conservative estimates, and are likely to be higher in the population^78^.

#### Computational modeling

The responses and response times in the 2AFC task were modeled using the Linear Ballistic Accumulator model LBA^40^. This model is developed as the simplest possible model for evidence accumulation in a decision-making task. It assumes that over the course of one choice, the participant’s evidence accumulation process can be approximated by a linear non-stochastic rise to a threshold value. The choice between various options follows from whichever linear process reaches the threshold value the soonest. Variability in responses and response times is accounted for by assuming variability across trials in the linear rise as well as the threshold value. This simple process accounts for many benchmark phenomena in (perceptual) decision making^40,55,79^, and yields comparable inferences about cognitive mechanisms to more complex decision making models^30,55,80^. The LBA model has five principle parameters, that may be constrained across conditions and accumulators: The evidence accumulation is characterized by a normal distribution with mean *v* and standard deviation *s*; The threshold is characterized by a uniform distribution [*B-A*, *B*] (i.e., *B* is the maximum threshold value, and *B-A* is the minimum). The model assumes a shift in the response time distribution that accounts for peripheral processes (typically referred to as *t*_0_).

To account for the hypothesized effect of core temperature on urgency, we included an additive term to the mean evidence accumulation *v*, such that the effective v = v_s_ + v_u_, that is, the sum of the stimulus-induced accumulation of evidence *v*_*s*_, and the temporal urgency signal *v*_*u*_ cf.^27,28^. Note that for the LBA model, the predictions of this parametrization are equivalent to a parametrization that implements a linearly decreasing threshold^8,9,15^, a proof of this equivalence is provided by Miletic and Van Maanen^38^. This makes our study not suitable for disentangling these theoretical proposals about the cognitive mechanism of urgency^11,16^.

To estimate posterior distributions of LBA parameters for each subject individually and at the group level simultaneously, we used differential evolution and Markov Chain Monte Carlo sampling with Metropolis-Hastings DE-MCMC^81,82^, as implemented in DMC^83^. Imposing a hierarchical structure between participant and group level parameters allows an individual participant’s parameters to be informed by the parameter values of all other participants. Individual participants’ deviations from group-level parameters are possible in so far such deviations are essential for a good fit^83^.

We parameterised the LBA such that we estimated *v*_*correct*_ and a *difference* parameter Δ*v* = *v*_*correct*_ − *v*_*error*_. Similarly, instead of estimating threshold *b*, we estimated the difference between threshold and upper bound of the start point distribution B = b − A. We fit three model parametrizations to the data, and selected the model that best balanced model flexibility and goodness-of-fit using a Bayesian Predictive Information Criterion (BPIC^41^). BPIC is preferred over BIC for hierarchically fitted models. These models either varied the drift rate (i.e., the sum of the stimulus-induced accumulation of evidence *v*_*s*_, and the temporal urgency signal *v*_*u*_, since these components cannot be disentangled in this design), or the choice threshold *B*, or both, across measurement moments and conditions. All other parameters (*A*, *s*_*error*_, *t*_0_, and Δ*v*) were estimated equal across conditions.

As a scaling constraint, the variability of the evidence accumulation process of correct choices was set to *s*_*correct*_ = 1. The measurement scale on which we optimized was seconds. RTs faster than 200 ms were excluded to allow convergence of the fitting routine (0.5% of the data, range: 0–6.5%, 17/27 participants had no such fast RTs).

For all parameters, we used wide, uninformed (uniform) priors. On the participant level, these were specified as follows for all conditions:

$$\begin{array}{lll}{v}_{correct} &  \sim  & U(\,-\,5,\,20)\\ {\rm{\Delta }}v &  \sim  & U(0,\,20)\\ {s}_{error} &  \sim  & U(0.1,\,10)\\ A &  \sim  & U(0.01,\,20)\\ B &  \sim  & U(0.01,\,20)\\ {t}_{0} &  \sim  & U(0.01,\,0.5)\end{array}$$

^1^

Group-level distributions are described by hypermeans and hyperSDs. Priors for the hypermeans were set to:

$$\begin{array}{lll}{v}_{correct} &  \sim  & U(0,\,10)\\ {\rm{\Delta }}v &  \sim  & U(0,\,10)\\ {s}_{error} &  \sim  & U(0.1,\,5)\\ A &  \sim  & U(0.01,\,10)\\ B &  \sim  & U(0.01,\,10)\\ {t}_{0} &  \sim  & U(0.01,\,0.5)\end{array}$$

For the hyperSDs, gamma priors were used:

$$\begin{array}{lll}{v}_{correct} &  \sim  & {\rm{\Gamma }}(1,\,1)\\ {\rm{\Delta }}v &  \sim  & {\rm{\Gamma }}(1,\,1)\\ {s}_{error} &  \sim  & {\rm{\Gamma }}(1,\,1)\\ A &  \sim  & {\rm{\Gamma }}(1,\,1)\\ B &  \sim  & {\rm{\Gamma }}(1,\,1)\\ {t}_{0} &  \sim  & {\rm{\Gamma }}(1,\,1)\end{array}$$

The number of chains *D* was set to three times the number of parameters for each model specification. Compared to other MCMC algorithms, DE-MCMC updates each chain’s state based on the difference between two other chains’ states (plus a random perturbation between [−0.001, 0.001]). This is known as cross-over. Cross-over weight was set to $2.38/\sqrt{D}$^81^ on the participant level, and randomly sampled from a uniform distribution between [0.5, 1] on the group level. Migration probability, was set to 0.05 during burn-in only.

A burn-in period of 1,000 iterations was followed by at least 5,000 iterations of sampling from the posterior, resulting in at least 300,000 samples from the posteriors. An immediate thinning factor of 5 was used to reduce computational load.

Chain convergence was assessed using the Gelman-Rubin diagnostic (median multivariate potential scale reduction factor <1.05 for all model specifications^84,85^) and visual inspection of the chain traces. For one participant, a minority of chains did not update states for many iterations (remained ‘stuck’), even with repeated restarts of the procedure. However, while inflating Gelman’s diagnostic, the influence of these stuck chains on the posterior distribution was minimal, and all other chains converged normally.

## Supplementary information

Supplementary Materials for: Core body temperature speeds up temporal processing and choice behavior under deadlines

## Data Availability

Data and model specifications are available upon request.

## Footnotes

1 Note that priors are not strictly necessary at the participant level. These priors were used only to define distributions from which starting points of the MCMCa chains were sampled.
