# Supplementary material for: Core body temperature speeds up temporal processing and choice behavior under deadlines
Source: Sci Rep. 2019 Jul 11;9:10053. doi: 10.1038/s41598-019-46073-3 (PMC6624282; doi:10.1038/s41598-019-46073-3)
Supplement: Supplementary file 1 — Supplementary Materials for: Core body temperature speeds up temporal processing and choice behavior under deadlines [file 41598_2019_46073_MOESM1_ESM.pdf]

Supplementary Materials for: Core body temperature speeds up temporal processing and choice behavior under deadlines

Leendert van Maanen,[1]*, Robbert van der Mijn,[2] Maurice H.P.H. van Beurden,[3] Linsey M.M. Roijendijk,[3] Boris, R.M. Kingma,[3] Steven Miletić,[1] & Hedderik van Rijn.[2]*

[1] Department of Psychology, University of Amsterdam
[2] Department of Psychology, University of Groningen
[3] Netherlands Organization for Applied Scientific Research, Unit Defense Safety and Security, Department of Training and Performance Innovations, Soesterberg, The Netherlands

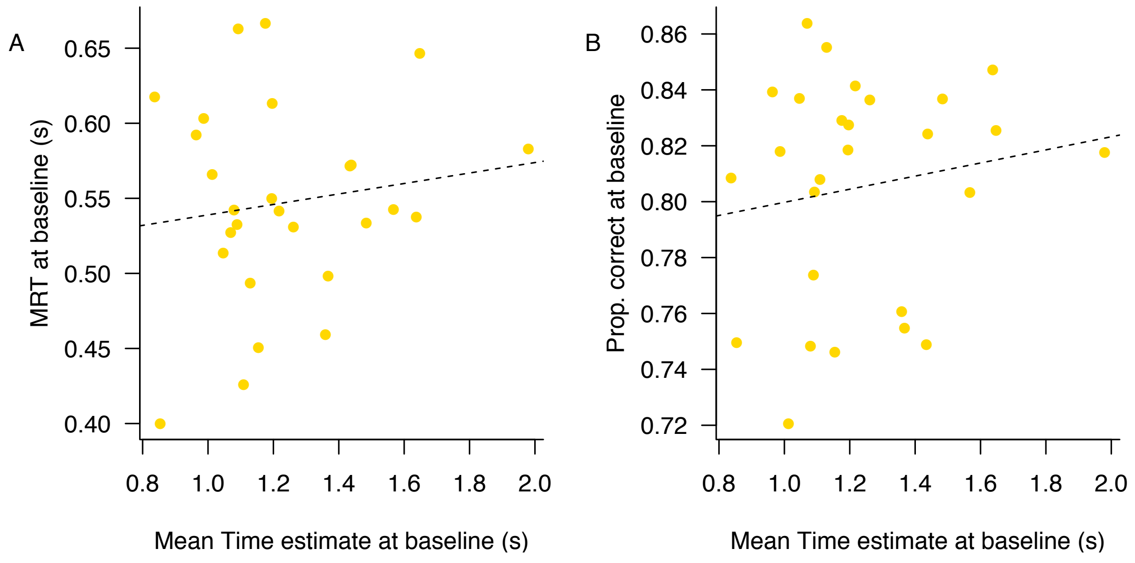

Figure S1. No evidence for a relationship between task behaviors at baseline. (A) mean response time (MRT) at baseline does not depend on mean time estimates at baseline. (B) mean accuracy at baseline does not depend on mean time estimates at baseline.

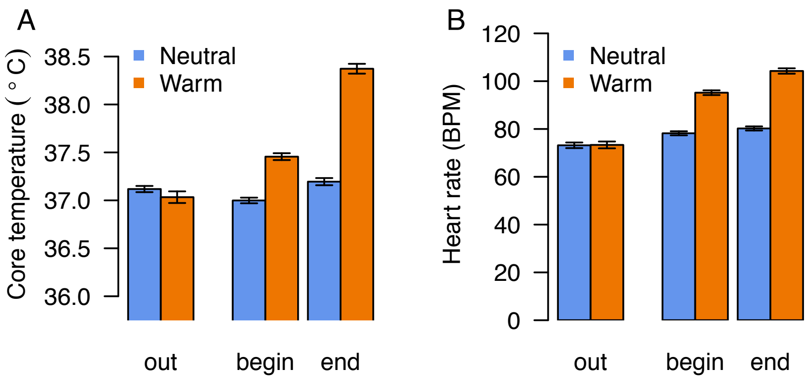

Figure S2. Manipulation check. The immersion of the participants in water with different temperatures differentially affected their core temperature (A) as well as their heart rate (B).

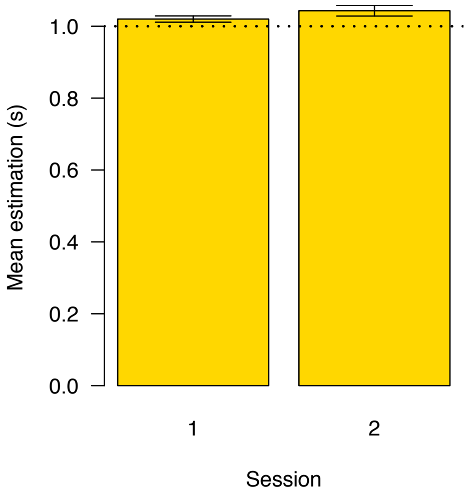

Figure S3. During a practice block, participants learned to time an interval of 1s. Independent of the session, participants were able to accurately reproduce the 1s interval in the practice block.

Table S1. Parameter estimates (mean and standard error of the mean) of the winning LBA model of the choice task (see text for abbreviations).

**Condition**	$v_{correct}$ **(se)**	v_{error} **(se)**	$s_{correct}$ **(se)**	s_{error} **(se)**	A **(se)**	B **(se)**	t_0 **(se)**
**Baseline 1**	4.51 (0.24)	2.46 (0.23)	1 (-)	1.63 (0.05)	1.41 (0.18)	1.46 (0.13)	0.09 (0.011)
**Baseline 2**	4.54 (0.24)	2.49 (0.23)	1 (-)	1.63 (0.05)	1.41 (0.18)	1.51 (0.14)	0.09 (0.011)
**36 Begin**	4.48 (0.23)	2.43 (0.23)	1 (-)	1.63 (0.05)	1.41 (0.18)	1.37 (0.13)	0.09 (0.011)
**36 End**	4.72 (0.29)	2.68 (0.28)	1 (-)	1.63 (0.05)	1.41 (0.18)	1.47 (0.17)	0.09 (0.011)
**38 Begin**	4.44 (0.23)	2.39 (0.22)	1 (-)	1.63 (0.05)	1.41 (0.18)	1.28 (0.12)	0.09 (0.011)
**38 End**	4.56 (0.26)	2.51 (0.25)	1 (-)	1.63 (0.05)	1.41 (0.18)	1.22 (0.14)	0.09 (0.011)

Supplementary Analysis 1. Skewness and conditional accuracy functions in the data.

The two theoretical accounts of time pressure (additive urgency, or a lower time-invariant threshold) make different predictions for the distribution of errors across response times, and for the shape of response time distributions. Specifically, the additive urgency hypothesis predicts relatively more errors at the end of the RT distribution (a so-called negative conditional-accuracy function), when time pressure increases, whereas a lower threshold hypothesis predicts an even distribution of errors across the distribution [1,2]. Another prediction from the additive urgency hypothesis is a stronger decrease in skewness of the RT distribution, when time pressure increases [3].

We tested these behavioral predictions, and found no evidence for the additive urgency hypothesis. Specifically, we fit a nonlinear logistic regression model (Equation 1) to the data of each condition and measurement moment separately, by minimizing the squared deviation using particle swarm optimization [4,5]. The model predicts the probability of a correct response as a function of RT, using four parameters, that each capture a specific pattern in the data. The crucial parameter here is *b*, capturing the downward slope of the conditional accuracy function. If *b* is higher, this indicates a stronger decrease in the slope [for details, we refer to 5].

$$f(x) = \frac{e^a}{e^a + e^{\left(b(x-d)+\frac{c}{x-d}\right)}} \qquad (1)$$

We found that – relative to baseline – *b* significantly differs between measurement moments ($F(1,26)=5.6$, $p=0.026$, Figure S4A), but not between conditions ($F(1,26)=2.6$, $p=0.11$), nor an interaction ($F<1$). This indicates that there is no evidence for a change in an additive urgency signal that is driven by the core temperature, only by measurement moment. In fact, the proportion of errors that are relatively slow actually decreases at the end of the tub session, independent of water temperature, which is inconsistent with the additive urgency hypothesis. A similar pattern is observed when analyzing skewness of the choice RT distribution: the End measurement shows more skewness then the Begin measurement ($F(1,26)=5.6$, $p=0.025$, Figure S4B), but there is no effect of condition nor an interaction ($Fs<1$).

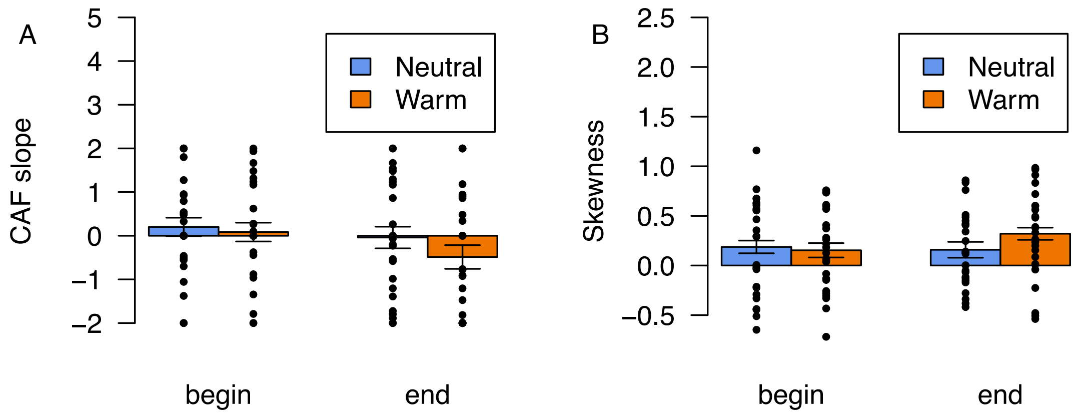

Figure S4. No evidence for additive urgency when alanyzing conditional accuracy functions (CAF) or skewness measures. (A) The slope of the CAF function in the choice task, relative to the baseline measure. (A) skewness of the choice response time distribution relative to the

baseline measure. Error bars indicate within-subject standard errors of the mean, dots indicate individual estimates.

Supplementary references

1. Karşılar, H., Simen, P., Papadakis, S. & Balci, F. Speed accuracy trade-off under response deadlines. *Front. Neurosci.* **8,** 248 (2014).
2. Frazier, P. & Yu, A. J. Sequential hypothesis testing under stochastic deadlines. *Adv. Neural Inf. Process. Syst.* **20,** 465–472 (2008).
3. Murphy, P. R., Boonstra, E. & Nieuwenhuis, S. Global gain modulation generates time-dependent urgency during perceptual choice in humans. *Nat. Commun.* **7,** 13526 (2016).
4. Clerc, M. *Particle swarm optimization*. (Wiley-ISTE, 2010).
5. Van Maanen, L., Katsimpokis, D. & Van Campen, D. Fast and Slow Errors: Logistic Regression to Identify Patterns in Accuracy-Response Time Relationships. *Behav Res Methods*
